# Heavy Metal Toxicity Following Metal‐on‐Metal Knee Arthroplasty Revision Surgery: A Case Report

**DOI:** 10.1155/cro/3777088

**Published:** 2026-11-09

**Authors:** Mohamad Assaf, Alexander Merckens, Solveig Tiepolt, Tom Lindner, Andreas Roth

**Affiliations:** ^1^ Department of Orthopedics Trauma and Plastic Surgery University of Leipzig Leipzig Germany uni-leipzig.de; ^2^ Department of Internal Medicine III Division of Nephrology University of Leipzig Leipzig Germany uni-leipzig.de; ^3^ Department of Nuclear Medicine University of Leipzig Leipzig Germany uni-leipzig.de

**Keywords:** chromium–cobalt intoxication, heavy metals, metallosis, neurotoxicity, quality of life, total knee replacement, wear

## Abstract

Metallosis is a rare complication; hence, previous investigations regarding this topic are restricted to case reports. Little has been published on metallosis following total knee arthroplasty (TKA). We report the case of a patient who experienced cobalt and chromium metallosis 14 years following TKA‐revision surgery. She developed a variety of symptoms that could indicate systemic clinical toxicity, including skin redness, headaches, irritability, cognitive decline, and sleep disturbances. Replacing the prosthesis, utilizing chelation therapy, and trials of double‐filtration plasmapheresis reduced the serum level of heavy metals upon regular laboratory follow‐ups, leading to a temporary relief of symptoms. The presence of confounding factors and other concomitant conditions makes the distinction between systemic metal exposure and suspected systemic clinical toxicity very challenging.

## 1. Introduction

Metallosis describes a local inflammatory reaction to metallic wear particles. In a minority of patients, systemic dissemination of cobalt (Co) and chromium (Cr) from corrosion leads to clinically relevant toxicity, most frequently reported after metal‐on‐metal hip arthroplasty [1–6]. Mechanical abrasion, fretting, and corrosion at modular junctions generate ions and nanoparticles that migrate from periprosthetic tissues into the circulation and may accumulate in distant organs [1, 5, 7–10].

Systemic Co and Cr toxicity may involve neurological, cardiovascular, hematologic, renal, and immunologic systems, often dissociating from circulating concentrations [2, 11]. It may even contribute to aseptic loosening of the implant itself [12]. We report a case of delayed, multisystem Co/Cr toxicity following revision total knee arthroplasty with a hinge construct, illustrating diagnostic complexity and therapeutic challenges.

## 2. Case History

We present the case of a female patient in her 50s who received a knee prosthesis due to posttraumatic knee osteoarthritis. Seven years following the primary implantation, the patiet underwent a revision surgery with a linked knee prosthesis. Managing this structural joint damage required multiple surgical procedures over time. In total, the patient underwent more than 40 knee procedures. Medical history included hay fever, house dust allergy, and nicotine dependence with approximately 20 cigarettes per day. The extensive clinical progression is graphically illustrated in Figure 1, and the Co and Cr measurements with major treatments are summarized in Table 1. The need for IRB approval was waived because this was an individual case report that did not include any personally identifiable information that could be traced back to the patient, nor did it involve any intervention.

**Figure 1:**
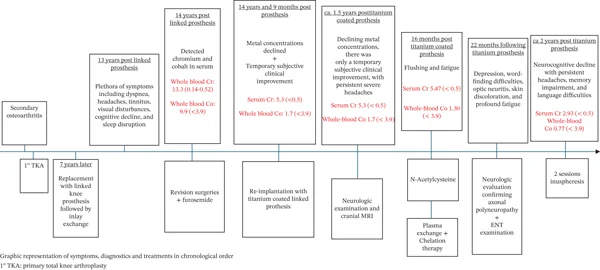
Graphical representation of the case description.

**Table 1:** Summary of cobalt and chromium measurements with major treatments.

Time	Chromium measurement		Cobalt measurement		Treatment
	Specimen type		Specimen type		
	Whole blood (ref 0.14–0.52 *μ*g/L)	Serum (*r* *e* *f* < 0.5 *μ*g/L)	Whole blood (*r* *e* *f* < 3.9 *μ*g/L)	Serum (*r* *e* *f* < 1.2 *μ*g/L)	
14 years following implantation of linked knee prosthesis	13.3		9.9		Revision: synovectomy and debridement
14.5 years following implantation of linked knee prosthesis	22.8		—		Prosthesis removal with cement‐spacer implantation + diuresis
1 month following explanation of linked knee prosthesis	7.01		3.9		
2 months following explanation → implantation of titanium‐nitride–coated linked knee prosthesis					
3 months following titanium‐coated prosthesis		5.3	1.7		
16 months following titanium‐coated prosthesis		5.47	1.3		Chelation therapy and diuresis
18 months following titanium prosthesis		4.4		1.9	
Nearly 2 years following titanium prosthesis + initiation of Inuspheresis		2.93		0.77	

Thirteen years following the implantation of the linked knee prosthesis, the patient developed a variety of unclear symptoms listed in Figure 1. The following year (i.e., nearly 14 years after implantation of the coupled knee prosthesis), laboratory testing revealed markedly elevated whole blood Cr 13.3 *μ*g/L (reference 0.14–0.52) and whole blood Co 9.9 *μ*g/L (reference < 3.9). Under the suspicion of prosthetic wear, revision surgery in February 2022 showed grayish synovitis consistent with metal corrosion (Krenn Type I). Since there were no signs of prosthesis loosening and the coupling mechanism showed only minor abrasion, synovectomy and debridement were performed. Histology confirmed wear‐induced neosynovialitis. Months later, when whole blood Cr rose to 22.8 *μ*g/L (ref 0.14–0.52), complete prosthesis removal with cement‐spacer implantation was undertaken. Intraoperatively, black bone discoloration was observed; histology revealed high‐grade particle‐induced neosynovialitis with abundant fine black granules, marked sclerosis, hypersensitivity‐related necroses, and fibrinous‐exudative reaction. Nephrological coassessment initiated a conservative approach with furosemide under strict fluid monitoring to enhance renal metal clearance. A couple of months later, Cr 7.01 *μ*g/L (whole blood) and Co 3.9 *μ*g/L (whole blood) had declined, with preserved renal function and enhanced urinary elimination (urine: Cr 10.6 *μ*g/L, Co 3.1 *μ*g/L).

After the two‐stage reimplantation with a titanium‐nitride–coated linked knee prosthesis, metal levels decreased further (serum Cr 5.3 *μ*g/L, ref < 0.5; whole blood Co 1.7 *μ*g/L, ref < 3.9; December 2022). Urine concentrations suggested enhanced elimination (urine Cr 20.0 *μ*g/L and urine Co 6.6 *μ*g/L). Despite declining metal concentrations, there was only a temporary subjective clinical improvement, with persistent severe headaches. A cranial MRI revealed no toxic or degenerative signal abnormalities were detected. Symptoms persisted, including tinnitus and cognitive decline. Serum Cr 5.47 *μ*g/L (ref < 0.5) and whole blood Co 1.30 *μ*g/L (ref < 3.9) remained detectable.

Because of continuing flushing and fatigue, oral N‐acetylcysteine (NAC) 1200 mg/day was initiated. The patient underwent several plasma exchange sessions (10 L cumulative), due to progressive symptoms with new gastrointestinal complaints, urticaria, and night sweats. This however provided transient relief. Despite ongoing chelation and enhanced urinary metal‐elimination (serum Cr 4.4 *μ*g/L, ref < 0.5; serum Co 1.9 *μ*g/L, ref < 1.2; urine Cr 11.8 *μ*g/L, urine Co 9.9 *μ*g/L), the patient developed word‐finding difficulties, optic neuritis, and skin discoloration. Neurological re‐evaluation showed axonal polyneuropathy with preserved blink reflexes, excluding trigeminal nerve injury. ENT examination found no objective correlate for persisting tinnitus. Finally, two Inuspheresis sessions (double‐filtration plasmapheresis) were performed to remove high‐molecular‐weight and protein‐bound metal complexes. Concurrent testing showed persistent but further reduced serum Cr 2.93 *μ*g/L (ref < 0.5) and serum Co 0.77 *μ*g/L (ref < 1.2). Clinically, headache and flushing improved transiently, but later recurred. [^18^F]FDG‐PET/CT (February 2025) revealed no metabolic correlate of Co/Cr neurotoxicity.

Throughout 2022–2025, renal function remained stable (creatinine and eGFR normal). Hematology showed transient iron‐deficiency anemia that normalized after substitution. Liver, thyroid, and glucose metabolism were unremarkable. Despite removal of all suspected sources and steadily declining systemic metal concentrations, symptoms continued, highlighting the complex and protracted course of Co and Cr toxicity.

## 3. Discussion

### 3.1. Pathophysiology in the Context of Hinge TKA

Metallosis is a rare but serious complication of joint arthroplasty, most frequently described after metal‐on‐metal hip replacements. In the present case, Co and Cr toxicity developed following a revision total knee arthroplasty using a linked hinge system originally designed for tumor reconstruction but nowadays also recommended for revision arthroplasty [13]. Such couplings, characterized by high constraint and less forgiving lubrication, are particularly prone to fretting, trunnion and hinge corrosion, and third‐body wear under long‐term mechanical stress [4, 5, 14, 15]. The underlying pathophysiology is multifactorial and involves a proinflammatory reaction with oxidative and mitochondrial stress leading to cell injury [1, 5, 16–18]. Histology in our case repeatedly showed abrasion‐type neosynovitis (Krenn Type I) with black pigment deposition, consistent with wear‐particle–driven inflammation [19].

Across metal‐on‐metal hip cohorts, Co and Cr typically rise during the first postoperative year (with some series showing a 6–12‐month peak) [20–22] and decline over subsequent years; long‐term trajectories are heterogeneous, with sustained decreases in well‐functioning resurfacings and late increases in a subset of patients [20–22]. Our patient′s presentation 14 years after revision underscores that delayed, low‐grade corrosion and depot off‐loading can sustain exposure long beyond the early postoperative window. Interestingly, there are no international cut‐off values for serum Co and Cr toxicity. A whole blood level of 7 *μ*g/L has been specified as a “concern level” by the Medicines and Healthcare products Regulatory Agency (UK MHRA) and is considered a gray zone by the European Hip Society and European Federation of National Associations of Orthopaedics and Traumatology. The highest whole blood level of Co and Cr in our patient reached 9.9 and 22.8 *μ*g/L, respectively (before the removal of the prosthesis).

Serial Co and Cr measurements should be interpreted as trends rather than absolute values. Co exhibits multiphasic retention kinetics with tissue half‐lives extending to several hundred days, while a substantial fraction of Cr is sequestered intracellularly within erythrocytes over their approximately 120‐day lifespan, thereby limiting both plasma‐based measurement and plasma‐based removal [17]. Consistent with previous reports, symptom severity in our patient dissociated from circulating metal concentrations [23, 24]; monitoring was therefore guided by clinical symptoms rather than by absolute metal levels.

### 3.2. Neurological and Neuropsychiatric Symptoms in Our Patient

Neurological involvement is frequently reported in arthroprosthetic Co/Cr exposure, including headache, cognitive impairment, tinnitus, visual disturbance, peripheral neuropathy, and neuropsychiatric symptoms [2, 3, 25].

Our patient experienced chronic right‐sided trigemino‐autonomic headache, tinnitus, cognitive decline, and mild axonal sensory polyneuropathy. Symptoms improved transiently after prosthesis exchange, but later recurred. In line with clinical reviews, auditory symptoms may improve after source removal, while peripheral neuropathy often persists despite falling Co concentrations [2]. Bridges et al. reported neurological symptoms at whole blood Co concentrations as low as 0.4 *μ*g/L [26], a finding consistent with the persistent neurocognitive complaints observed in our patient despite serum Co levels below 1 *μ*g/L. Brain MRI revealed a right postcentral AVM/DVA with cavernoma in our patient, but no metal‐typical signals; [^18^F]FDG‐PET/CT showed no cortical hypometabolism, contrasting with quantitative hypometabolism patterns reported in symptomatic cobaltism cohorts [26].

### 3.3. The Potential Role of Hypersensitivity

Metal debris may trigger lymphocyte‐predominant synovial reactions (aseptic lymphocyte‐dominated vasculitis‐associated lesions [ALVALs]) and less commonly systemic hypersensitivity such as urticaria or dermatitis, even at moderate exposure levels [1, 27–29]. ALVAL severity does not correlate reliably with metal ion concentrations, underscoring an immune‐mediated component distinct from simple dose effects [30, 31]. In our patient, flushing, erythema, and musculoskeletal pain preceded the diagnosis and were initially attributed to histamine intolerance. Synovial histology repeatedly demonstrated an abrasion‐type (Krenn Type I) membrane rather than ALVAL, yet the systemic phenotype (vasomotor/allergic‐like symptoms) is compatible with hypersensitivity‐amplified expression in a susceptible host.

Formal Co/Cr allergy testing (patch or LTT) was not performed, limiting definitive attribution of a systemic hypersensitivity mechanism in the case of our patient. Metal debris generated by prosthetic wear comprises both dissolved Co/Cr ions and Co–Cr nanoparticles, which differ substantially in their biological effects. Experimental data indicate that nanoparticles exceed ionic Co in their capacity to induce oxidative stress and neurotoxicity [32]. Moreover, nanoparticulate deposits within periprosthetic tissues are difficult to eliminate by synovectomy alone and thus constitute a persistent source of low‐grade metal release long after implant exchange.

Furthermore, the patient had multiple potential contributors to her symptom profile, including PTSD, recurrent depression, histamine intolerance, nicotine dependence (approximately 20 cigarettes/day), and structural knee complications. Each could amplify systemic symptoms such as fatigue, flushing, or cognitive complaints, complicating causal attribution [30]. Chronic smoking in particular is associated with modestly elevated baseline Co and Cr levels in general‐population biomonitoring [33] and may prime oxidative and immune pathways but does not account for the sustained systemic elevations once the implant was removed.

Based on all the above, hypersensitivity can be considered as a potential cause for the symptoms experienced by our patient, but not a confirmed diagnosis.

### 3.4. Treatments Tried and Outcome

#### 3.4.1. Source Control

Removal of the mechanical source of corrosion with meticulous synovectomy remains the cornerstone of management and the clearest and most durable clinical/biochemical improvement after source control [2, 3, 5]. In this case, the corroded hinge construct was explanted (July 2022) and replaced with a TiN‐coated device; subsequent cerclage removal (January 2025) coincided with stepwise declines in circulating metals, consistent with mechanical source control.

#### 3.4.2. Renal Handling

The kidneys are the principal organs responsible for the elimination of Co and Cr. With normal creatinine/eGFR, metal levels usually reflect ongoing input from implants or depots rather than renal retention, matching our patient′s course [34].

In line with the concept of enhanced renal elimination, forced diuresis was attempted initially in our patient, followed by chelation with NAC to further increase renal elimination. Case‐level evidence shows reductions in blood Co/Cr with NAC, yet benefits are adjunctive and not curative in the presence of tissue/RBC depots [2, 35]. Despite increased urinary excretion and only slightly elevated levels for Cr and Co, symptom improvement was only transient in our patient.

#### 3.4.3. Extracorporeal Approaches

Because symptoms persisted in our patient, extracorporeal removal was escalated to therapeutic plasma exchange and later double‐filtration plasmapheresis (Inuspheresis). Case reports/series show variable biochemical responses with short‐lived clinical relief. For example, plasma exchange can reduce Co by ~two‐thirds acutely with rebound to baseline within hours, typical of depot off‐loading without definitive source control [36].

The patient′s fluctuating symptoms despite decreasing levels illustrate the difficulty of managing depot off‐loading over time [36]. Several authors note poor correlation between Co/Cr concentrations and clinical severity, emphasizing that symptom dynamics often reflect redistribution and tissue interactions rather than circulating levels [23, 24].

## 4. Summary

Metallosis resulting from metal alloy implants in total knee replacement is rare but can have debilitating effects on the patient. It is also important that laboratory values are checked repeatedly. Even a clinically and radiologically unremarkable prosthesis can, if its design permits, be associated with massive abrasion and metallosis and its consequences. While replacing the implant and attempting to lower the serum level of the heavy metals can contribute to a temporary improvement of the symptoms, the adverse effects seem to rebound and the low quality of life persists.

In the case of our patient, not every symptom could be linked to metal toxicity. Additionally, many of the diagnostics failed to reveal evidence of systemic clinical toxicity. The presence of confounding factors and other concomitant conditions makes the distinction between systemic metal exposure and suspected systemic clinical toxicity very challenging. Clinical assessment should therefore address the symptoms as well as metal concentrations, and other conditions must still be ruled out.

Studies are needed to examine the incidence and risk factors of metallosis following joint replacement surgeries and to identify the population of patients most susceptible to developing metallosis.

## Funding

No funding was received for this manuscript.

## Ethics Statement

The authors have nothing to report.

## Conflicts of Interest

The authors declare no conflicts of interest.

## Data Availability

No new data were created or analyzed in this study. Data sharing is not applicable to this article.
